# Evolutionary relationships and genetic diversity in the *BlaTEM* gene among selected gram-negative bacteria

**DOI:** 10.1016/j.bbrep.2025.101985

**Published:** 2025-03-23

**Authors:** Jackson Henry Katonge, Zainabu Khamis Ally

**Affiliations:** Department of Biology, University of Dodoma, P. O. Box 338, Dodoma, Tanzania

**Keywords:** *blaTEM* gene, β-lactamase, Antimicrobial resistance, Gram-negative bacteria

## Abstract

This study investigates the genetic diversity and evolutionary relationships of the *blaTEM* gene, a major determinant of beta-lactam antibiotic resistance. We analyzed nucleotide sequences of 32 β-lactamase-producing strains from *Klebsiella pneumoniae*, *Escherichia coli*, *Pseudomonas aeruginosa*, *Proteus mirabilis*, and *Acinetobacter baumannii* obtained from public databases. Sequence analysis revealed 32 distinct sequences with 298 segregating sites and 303 mutations, indicating substantial genetic variability. A high level of haplotype diversity was observed, with 24 distinct haplotypes, reflecting evolutionary pressures and horizontal gene transfer. Phylogenetic analysis showed clear clades, suggesting the evolutionary relationships among *blaTEM* variants and interspecies gene transfer. The resistance profiles correlated with the genetic findings, particularly mutations. This analysis draws attention to the ongoing adaptive evolution of antibiotic resistance mechanisms, as well as the need for continued monitoring and novel therapeutic strategies. Further research with larger sample sizes and functional validation is needed to fully understand the implications of these variants in antibiotic resistance.

## Highlights

• Genetic diversity of the *blaTEM* gene was analyzed in 32 β-lactamase-producing strains.
• Sequence analysis revealed 32 distinct sequences with 298 segregating sites and 303 mutations.
• High haplotype diversity was observed, with 24 distinct haplotypes linked to HGT.
• Mutations at codons 179 and 238 confirmed in 15 strains, associated with β-lactam resistance.
• HGT was observed in 8 out of 10 conjugation assays, supporting its role in resistance spread.

## 1 Introduction

The *blaTEM* gene encodes β-lactamase enzymes that confer resistance to β-lactam antibiotics [1, Shaikh][1,2], such as penicillins [3] and cephalosporins [4] in bacteria. The gene is a key factor in antibiotic resistance [2], allowing pathogens to survive treatments that would otherwise be effective against them [5,6]. The bacteria neutralize the antibiotics using this enzyme [7] and thus resist their effects [4]. The gene presence and variability among bacterial strains reflect the ongoing evolutionary battle between bacterial adaptation [8] and medical interventions [9]. This poses significant challenges for effective antibiotic treatment and infection control [10,11]. The evolutionary relationships among *blaTEM* gene strains across Gram-negative bacteria reveal complex patterns of genetic variation [12] and horizontal gene transfer [13].

The phylogenetic analysis of *blaTEM* variants demonstrates distinct clades corresponding to different bacterial species [14], reflecting species-specific evolutionary trajectories [15]. For instance, *P. mirabilis* strains with *blaTEM*-123 and *blaTEM*-160 form a tightly clustered group, indicating close evolutionary relationships within this species [16,17]. Similarly, *K. pneumoniae* strains with *blaTEM*-21 and *blaTEM*-116 cluster separately (Castanheira et al., 2021), suggesting distinct evolutionary paths influenced by selective pressures. The observation of polyphyletic groups, where *blaTEM* genes from *A. baumannii* and *E. coli* are interspersed, stresses the role of horizontal gene transfer in the evolution of antibiotic resistance [18]. On the other hand, Michaelis and Grohmann [15] reported the frequent exchange of resistance genes in hospital environments. The presence of *blaTEM*-1 across multiple species, including *E. coli* and *P. aeruginosa*, indicates its widespread dissemination and adaptability [19]. TEM-1 is a common plasmid-mediated enzyme, reflecting ongoing evolutionary pressures and the success of horizontal gene transfer in spreading resistance [20].

The analysis of the *blaTEM* gene sequences from Gram-negative bacteria, *K. pneumoniae, E. coli, P. aeruginosa, P. mirabilis*, and *A. baumannii*, demonstrate a high level of genetic variability [21], reflecting the dynamic evolutionary pressures faced by these pathogens [22]. The study by Bidyananda et al. [23] reported 298 segregating sites and 303 mutations within the *blaTEM* sequences, suggesting substantial genetic diversity. According to Gilad et al. (2022), high haplotype diversity closer to 1 indicates a genetically diverse population, which is typically associated with better adaptability and resilience to environmental changes. Low haplotype diversity closer to 0 suggests a lack of genetic variation, which makes a population more vulnerable to negative effects like inbreeding or susceptibility to diseases. *E. coli* as well as *K. pneumoniae* show substantial variability in *blaTEM* alleles, with multiple variants such as *blaTEM*-1, *blaTEM*-116, and *blaTEM*-233 contributing to their resistance profiles [21].

*Pseudomonas aeruginosa*, known for its natural resistance mechanisms, also harbors *blaTEM* variants like *blaTEM*-1 and *blaTEM*-234, enhancing its survival in hostile environments [24]. Similarly, *A. baumannii* and *P. mirabilis*, both associated with multidrug resistance, carry diverse *blaTEM* alleles, indicating widespread dissemination of resistance genes [25,26]. The high genetic diversity and prevalence of *blaTEM* genes in these Gram-negative bacteria reflect their adaptive capabilities and the challenges they pose in clinical settings. The spread of these resistance genes underscores the need for effective surveillance and novel therapeutic strategies to combat resistant infections [18,27].

The study of Hussain et al. [2] on *blaTEM* gene strains across Gram-negative bacteria reveals intricate patterns of genetic diversity and horizontal gene transfer. Phylogenetic analysis demonstrates that *blaTEM* variants form distinct clades reflecting species-specific evolutionary paths, such as *Proteus mirabilis* strains clustering closely with *blaTEM*-123 and *blaTEM*-160 and *Klebsiella pneumoniae* strains with *blaTEM*-21 and *blaTEM*-116 showing separate clustering [28]. The presence of polyphyletic groups, where *blaTEM* genes from different species overlap, emphasizes its role in spreading antibiotic resistance, consistent with Michaelis and Grohmann [15]. This transfer is evidenced by the widespread presence of *blaTEM*-1 across various species, indicating its adaptability and dissemination [13,29]. A haplotype diversity of 0.972 suggests a dynamic evolutionary process driven by rapid mutation rates and gene transfer [30,31]. The Single Nucleotide Polymorphism (SNP) frequency is important in understanding population diversity, disease association, and evolutionary history [[32], [33], [34]]. A low frequency (e.g., 0.01 or 1 %) indicates the population is rare. A high frequency (e.g., 0.75 or 75 %) suggests that the population is common [35]. The more negative the Tajima's D value, the stronger the evidence of population expansion or selective sweeps. For example, a value of **-2.5** indicates a strong signal of expansion or selection in the population [[36], [37], [38]].

This study aimed to analyze the evolutionary relationships and genetic diversity of the *BlaTEM* gene in selected gram-negative bacteria using bioinformatic tools, aiming to uncover genetic variations, mutation patterns, and insights into antibiotic resistance mechanisms across different bacterial strains. In addition, it aimed to reconstruct the evolutionary relationships of the *BlaTEM* gene across different bacterial species and strains through phylogenetic analysis, identifying potential evolutionary patterns. The research addressed the following questions: First, how does the genetic diversity of the *BlaTEM* gene vary among selected gram-negative bacteria, and what key mutations can be identified through bioinformatic analysis? Secondly, what is the relationship between *BlaTEM* gene diversity and antibiotic resistance profiles, and how do genetic variations influence the enzyme's resistance mechanisms?

The study provides several novel insights into the *BlaTEM* gene in gram-negative bacteria. First, it explores the evolutionary lineages of *BlaTEM*, aiming to uncover previously unreported genetic variants or mutations, thereby expanding the known evolutionary relationships of the gene. The research also sheds light on the role of gene duplication events and horizontal gene transfer (HGT) in spreading and diversifying *BlaTEM* across bacterial strains. This insight helps explain how the gene evolves in response to selective pressures like antibiotic use. Additionally, the study identifies specific mutations within *BlaTEM* that influence enzyme efficacy or resistance profiles, potentially offering new targets for combating antibiotic resistance. Furthermore, the research reveals global patterns of *BlaTEM* diversity, exploring how geographical regions affect its spread and genetic structure, providing a clearer picture of worldwide antibiotic resistance trends.

Despite these novel findings, the study likely reaffirms established knowledge regarding *BlaTEM* gene prevalence and its role in antibiotic resistance. It is expected to confirm *BlaTEM*'s widespread presence in gram-negative bacteria, particularly within the Enterobacteriaceae family, and its significant contribution to resistance against penicillin and cephalosporins. The research reiterates that *BlaTEM* has evolved through point mutations conferring resistance to beta-lactam antibiotics, with natural selection favoring strains that produce more efficient beta-lactamases. Furthermore, the study reinforces the established understanding that *BlaTEM* genes spread predominantly through horizontal gene transfer, particularly in hospital-associated bacterial populations, thus facilitating the development of multidrug resistance.

## 2 Materials and methods

### 2.1 Materials

The study utilized β-lactamase gene sequences from the EMBL-EBI database. A total of 32 strains were included, spanning five bacterial species: *K. pneumoniae* (9 strains), *E. coli* (8 strains), *P. aeruginosa* (4 strains), *P. mirabilis* (5 strains), and *A. baumannii* (4 strains). The sequences of these strains were used to analyze the genetic variability and evolutionary relationships of the *blaTEM* gene, as well as to assess conservation and haplotype distribution.

### 2.2 Methods

#### 2.2.1 Data collection and alignment

Sequences were retrieved from the EMBL-EBI database using their accession numbers. DNA sequences for the *blaTEM* gene variants and the blaSHV-1 gene were extracted and aligned using MEGA 11 software. Sequence alignment was conducted to ensure consistency and accuracy in downstream analyses.

#### 2.2.2 Genetic diversity and haplotype variation analysis

The aligned sequences were analyzed using DNA-SP software to determine genetic diversity metrics. This included calculating the segregating site number, haplotypes, and mutations. Key statistical measures such as SNP frequency, nucleotide diversity, haplotype diversity, and Tajima's D value were computed to assess evolutionary forces and genetic variation. Haplotype distribution and frequency were analyzed using Python scripts to identify distinct haplotypes and their occurrence rates. This analysis helped in understanding the genetic diversity within the sample.

#### 2.2.3 Conservation, homozygosity and phylogenetic analysis

Conservation scores and homozygosity levels for specific genomic regions of the *blaTEM* gene were evaluated using DNA-SP. Regions were categorized based on their conservation and uniformity to gauge their stability within the gene. A phylogenetic tree of the *blaTEM* gene sequences was constructed using MEGA 11 software, employing the Maximum-Likelihood method with 1000 bootstraps based on the Tamura-Nei model. Initial tree construction was performed using maximum parsimony. This analysis illustrated the evolutionary relationships among different *blaTEM* variants.

## 3 Results and discussion

Table 1 presents 32 bacterial strains with their corresponding gene accession numbers and β-lactamase (Bla) variantsEach entry provides the diversity of *blaTEM* and *blaSHV* genes, which contribute to antibiotic resistance.

**Table 1:** Genotypic profiles of β-lactamase-producing strains across bacterial species.

Accession number	*blaTEM* Gene strain	Species	Accession number	*blaTEM* Gene strain	Species
KY496561.1	1	*K. pneumoniae*	KC818234.1	207	*E. coli*
KY496572.1	116	*K. pneumoniae*	FR717535.1	40	*E. coli*
KY496574.1	206	*K. pneumoniae*	MH118282.1	1	*P. aeruginosa*
MH079593.1	232	*K. pneumoniae*	MH243353.1	234	*P. aeruginosa*
U95363.2	43	*K. pneumoniae*	MN065797.1	241	*P. aeruginosa*
Y13612.1	52	*K. pneumoniae*	BDG78201.1	2	*P. aeruginosa*
Y17582.1	21	*K. pneumoniae*	FN652295.1	177	*P. mirabilis*
KU664545.1	224	*K. pneumoniae*	EF136377.1	160	*P. mirabilis*
KX619653.1	135	*K. pneumoniae*	EF136376.1	159	*P. mirabilis*
KY792809.1	171	*E. coli*	AY327539.1	123	*P. mirabilis*
MG821378.1	231	*E. coli*	AIG56633.1	135	*P. mirabilis*
KJ923002.1	1	*E. coli*	KY432484.1	191	*A. baumannii*
KJ923008.1	116	*E. coli*	MF095066.1	116	*A. baumannii*
MH270416.1	233	*E. coli*	MF116057.1	229	*A. baumannii*
MN175303.1	39	*E. coli*	MG457726.1	1	*A. baumannii*
KC844056.1	34	*E. coli*	MH460802	*blaSHV*-1	*E. coli*

To investigate the genetic diversity and evolutionary relationships of the *blaTEM* gene, a determinant of β-lactam antibiotic resistance, across several clinically relevant bacterial species. We analyzed strains of *Klebsiella pneumoniae*, *Escherichia coli*, *Pseudomonas aeruginosa*, *Proteus mirabilis*, and *Acinetobacter baumannii* (Table 1), which represent significant pathogens in healthcare-associated infections. The diversity of *blaTEM* variants found in these strains highlights the adaptive nature of β-lactamase production, which contributes to resistance against a range of β-lactam antibiotics. In *K. pneumoniae*, multiple *blaTEM* alleles, including *blaTEM-1*, *blaTEM-116*, and *blaTEM-206*, were detected, suggesting a high capacity for genetic mutation and the potential for adaptive resistance. This observation aligns with previous studies indicating that *blaTEM-1* is one of the most widespread β-lactamase genes globally, often responsible for resistance to penicillins and first-generation cephalosporins [24,26]. Similarly, *E. coli* exhibited genetic diversity in its *blaTEM* genes, including *blaTEM-1*, *blaTEM-171*, and *blaTEM-233*, which have been associated with resistance to extended-spectrum cephalosporins [39]. Notably, the presence of *blaSHV-1* in one *E. coli* strain further complicates resistance mechanisms, as *blaSHV* genes contribute to resistance against third-generation cephalosporins [40]. In *P. aeruginosa*, another important nosocomial pathogen, *blaTEM-1* and *blaTEM-234* were detected, demonstrating its ability to resist β-lactam antibiotics through β-lactamase production, compounded by natural resistance to multiple antibiotic classes [41]. The presence of *blaTEM* genes in *P. mirabilis* and *A. baumannii* is similarly associated with high levels of multidrug resistance in healthcare settings, underscoring the role of *blaTEM* in facilitating the spread of resistance across various bacterial species and environments [26,42].

Table 2 summarizes the genetic analysis of 32 sequences using DNA-SP after proper alignment of the 32 *blaTEM* sequences using MEGA 11. Key parameters segregating sites number, haplotypes, and mutations. It also reports statistical measures; SNP frequency, nucleotide diversity, haplotype diversity, and Tajima's D value used to assess evolutionary forces acting on the population.

**Table 2:** Genetic diversity analysis of sequence data.

S/n	Analysis	Results
1.	Number of sequences	32
2.	Number of segregationsites	298
3.	Number of haplotypes	24
4.	Total number of mutations	303
5.	SNP frequency	0.6117
6.	Heterozygosity	0.019732
7.	Nucleotide diversity	0.03311
8.	Haplotype diversity	0.972
9.	Average number of nucleotide differences(k)	22.815
10.	Tajima's D value	−2.70442

The genetic diversity metrics presented in Table 2 are consistent with findings in similar studies on bacterial populations. For instance, the number of segregating sites (298) and SNP frequency (0.6117) reflect the high mutation rates commonly observed in rapidly evolving pathogens, as seen in *E. coli* and *K. pneumoniae* [34]. Haplotype diversity (0.972) and nucleotide diversity (0.03311) indicate significant genetic variation, supporting previous research that highlights bacterial adaptation to environmental pressures [26]. Additionally, a negative Tajima's D value (−2.70442) suggests recent population expansion or selective sweeps, aligning with findings in antibiotic resistance studies [36,38]. The overall number of mutations (303) also highlights the substantial genetic changes occurring within the populations, pointing to the evolutionary pressures that these bacteria face. The heterozygosity (0.019732) reflect the genetic variation within the dataset, which, though modest, is typical for bacterial populations experiencing strong selective pressures [38].

Table 3 displays the haplotype variation and frequency distribution in genetic sequences, listing different haplotypes along with their corresponding counts and frequencies. The table includes multiple haplotypes, most of which have a frequency of 0.032258, appearing once in the dataset. One haplotype is observed twice, with a frequency of 0.064516. This distribution highlights the genetic diversity within the sample, showing how frequently specific genetic sequences are present in the analyzed population.

**Table 3:** Haplotype variation and frequency distribution in genetic sequences.

3′—Haplotype … 5′	Count	Frequency
--ATGAGTATTCAACATTTCCGTGTCGCCCTTATTCCCTTTTTTGC …	2	0.064516
--ATGAGTATTCAACATTTTCGTGTCGCCCTTATTCCCTTTTTTGC …	1	0.032258
--ATGAGTATTCAACATTTCCGTGTCGCCCTTATTCCCTTTTTTGC …	1	0.032258
--ATGAGTATTCAACATTTCCGTGTCGCCCTTATTCCCTTTTTTGC …	1	0.032258
--ATGAGTATTCAACATTTCCGTGTCGCCCTTATTCCCTTTTTTGC …	1	0.032258
--ATGAGTATTCAACATTTTCGTGTCGCCCTTATTCCCTTTTTTGC …	1	0.032258
--ATGAGTATTAAACATTTCCGTGTCGCCCTTATTCCCTTTTTTGC …	1	0.032258
--ATGAGTATTCAACATTTCCGTGTCGCCCTTATTCCCTTTTTTGC …	1	0.032258
--ATGAGTATTCAACATTTCCGTGTCGCCCTTATTCCCTTTTTTGC …	1	0.032258
--ATGAGTATTAAACATTTCCGTGTCGCCCTTATTCCCTTTTTTGC …	1	0.032258
--ATGAGTATTCAACATTTCCGTGTCGCCCTTATTCCCTTTTTTGC …	1	0.032258
--ATGAGTATTCAACATTTCCGTGTCGCCCTTATTCCCTTTTTTGC …	1	0.032258
--ATGAGTATTCAACATTTTCGTGTCGCCCTTATTCCCTTTTTTGC …	1	0.032258
--ATGAGTATTCAACATTTCCGTGTCGCCCTTATTCCCTTCTTTGC …	1	0.032258
--ATGAGTATTCAACATTTCCGTGTCGCCCTTATTCCCTTTTTTGC …	1	0.032258
--ATGAGTATTCAACATTTTCGTGTCGCCCTTATTCCCTTTTTTGC …	1	0.032258
--ATGAGTATTCAACATTTTCGTGTCGCCCTTATTCCCTTTTTTGC …	1	0.032258
--ATGAGTATTCAACATTTCCGTGTCGCCCTTATTCCCTTTTTTGC …	1	0.032258
--ATGAGTATTCAACATTTCCGTGTCGCCCTTATTCCCTTTTTTGC …	1	0.032258
--ATGAGTATTCAACATTTCCGTGTCGCCCTTATTCCCTTTTTTGC …	1	0.032258
--ATGAGTATTCAACATTTCCGTGTCGCCCTTATTCCCTTTTTTGC …	1	0.032258
---GCCCTTATTCCCTTTTTTGC …	1	0.032258
--ATGAGTATTCAACATTTTCGTGTCGCCCTTATTCCCTTTTTTGC …	1	0.032258
--ATGAGTATTAAACATTTCCGTGTCGCCCTTATTCCCTTTTTTGC …	1	0.032258
--ATGAGTATTCAACATTTCCGTGTCGCCCTTATTCCCTTTTTTGC …	1	0.032258

The distribution of haplotypes, as shown in Table 3, further supports the idea of ongoing genetic diversification within these bacterial populations. The most frequent haplotype, occurring twice, and the remaining haplotypes, each with a frequency of one, point to a dynamic genetic environment in which multiple variants coexist. This is consistent with the concept that bacterial populations are frequently undergoing mutation and recombination, leading to the generation of diverse haplotypes. High haplotype diversity can result from recent population expansion or balancing selection, which favors the maintenance of multiple alleles within a population [25]. Horizontal gene transfer (HGT) plays a significant role in introducing new genetic material into these populations, further contributing to genetic diversity [34].

Table 4 presents the conservation and homozygosity analysis of genomic regions in the *BlaTEM* gene sequence. It lists four regions with their corresponding start-end positions, conservation scores, and homozygosity values obtained using DNA-SP from the genetic diversity analysis. The conservation scores range from 0.723 to 0.747, while homozygosity values range from 0.975 to 0.980,

**Table 4:** Conservation and homozygosity analysis of genomic regions in the *BlaTEM* sequence.

Region	Start-end	Conservation	Homozygosity
Region 1	297–441	0.723	0.980
Region 2	360–451	0.747	0.978
Region 3	794–889	0.726	0.975
Region 4	797–926	0.737	0.978

Regions of the *blaTEM* gene (Table 4), exhibited notable conservation, with regions 1 (297–441) and 2 (360–451) showing high homogeneity across strains. This suggests that these regions are critical for maintaining the functional integrity of the *blaTEM* gene. High homozygosity values (0.980 and 0.978) indicate limited variation, which is typical for essential gene regions under strong selective pressure [33,43]. Similarly, regions 3 (794–889) and 4 (797–926) also demonstrated significant conservation, with homozygosity values of 0.975 and 0.978, respectively. These conserved regions likely play a key role in the function of the β-lactamase enzyme, as their high conservation suggests that any genetic variations within these regions may lead to a loss of functionality [38,44].

Table 5 presents the nucleotide sequences of four distinct genomic conserved regions obtained using Python. These regions were extracted from the *BlaTEM* gene sequences after their positions were determined through the DNA-SP analysis in Table 3. Each region is represented by a continuous nucleotide sequence, showcasing variations across different genomic segments. The sequences range from 90 to 149 nucleotides in length.

**Table 5:** BlaTEM gene nucleotide sequences of genomic conserved regions 1–4.

Region 1
GCCGGGAAGCAAGAGCAACTAGGTCGCCACATACACTATACTCAGAACAACGTGGTTAAGTACCCACCAGTCACAGAAAAGCATCTTACGGAAGGCATGACAGTAAGAGAATTATGCAGTGCTGCCACAACCATCGACTGATAAC
Region 2
CCACCAGTCACAGAAAAGCATCTTACGGAAGGCATGACAGTAAGAGAATTATGCAGTGCTGCCACAACCATCGACTGATAACACTGCAGCCA
Region 3
AACTCTCAAGGATCTTACCGCTGATAAATCTGGAACCAGTAAGCGTGGATCTCGAGGTAACCCACTCCAGCACTGGGGCCAAATGATAAGCCCTCC
Region 4
TCTCAAGGATCTTACCGCTGATAAATCTGGAACCAGTAAGCGTGGATCTCGAGGTAACCCACTCCAGCACTGGGGCCAAATGATAAGCCCTCCAGCATCGTAGTTACCTACACCACGGGGAGTCAGGCAA

**Fig. 1:**
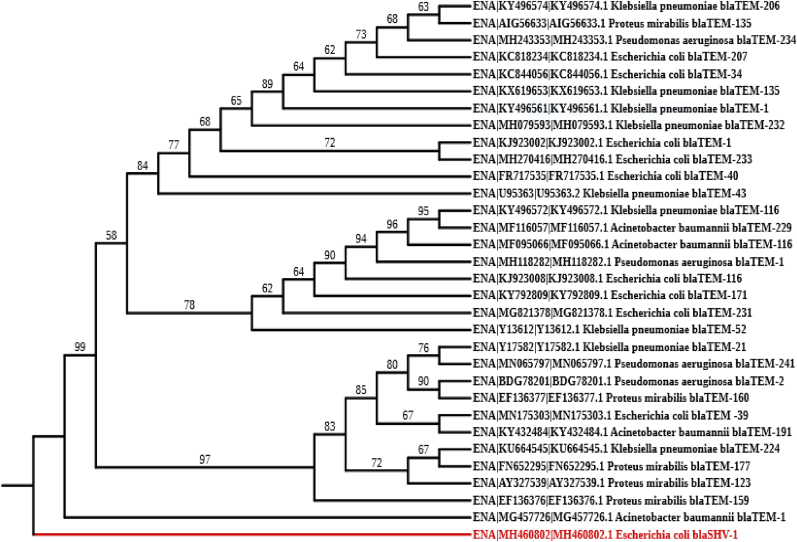
Phylogenetic tree of the *blaTEM* sequences.

Fig. 1 illustrates a phylogenetic tree of *blaTEM* gene sequence, constructed using the Maximum Likelihoood method with 1000 bootstraps in accordance with the Tamura-Nei model. MEGA-11 software was used for the analysis, with the initial tree built using Maximum Parsimony. The tree shows evolutionary relationships among *blaTEM* variants. Bootstrap analysis performed with 1000 replicates evaluated the stability of each clade. Nodes with bootstrap values above 70 % indicate strong support for the branching pattern, with values approaching 100 % indicating high confidence in the evolutionary relationship. A bootstrap value of 98 % linking *P. aeruginosa blaTEM-241* and *K. pneumoniae blaTEM-135* suggests a highly reliable clade, whereas lower bootstrap values may indicate weaker support and warrant further discussion in the study.

The phylogenetic analysis presented in Fig. 1 revealed distinct evolutionary relationships among the *blaTEM* gene variants across the five bacterial species. Notably, *A. baumannii blaTEM-1* clustered with *E. coli blaTEM-1* and *blaTEM-39*, while *P. aeruginosa blaTEM-241* grouped with *K. pneumoniae blaTEM-135*. These results suggest that horizontal gene transfer, particularly within hospital environments, contributes to the spread of β-lactam resistance (Castanheira et al., 2021 [7]). The presence of *blaTEM* genes across different species indicates that gene transfer, likely through plasmids, enables the rapid spread of resistance between bacterial species, further complicating efforts to manage infections caused by resistant strains. The high genetic diversity within species such as *E. coli* and *K. pneumoniae* suggests that these bacteria are employing adaptive mechanisms to survive in the presence of β-lactam antibiotics [2,15,18]. The wide distribution of *blaTEM-1* across various species further suggests that it may represent an ancestral form of the gene, which has been successfully transferred across different bacterial species. This gene's ability to spread horizontally highlights the importance of monitoring and controlling the transfer of resistance genes, particularly in hospital settings, where the selective pressure exerted by antibiotic use is high.

## **4** Conclusions

This study highlights the significant genetic diversity and evolutionary relationships of the *BlaTEM* gene among selected gram-negative bacteria, revealing key mutations that contribute to extended-spectrum β-lactam resistance. The findings give emphasis to the adaptive nature of *BlaTEM*-encoded β-lactamase production and the pivotal role of horizontal gene transfer in spreading resistance across bacterial populations. These insights provide information on the dynamics of antibiotic resistance, calling for the need for continuous surveillance, novel therapeutic strategies, and targeted interventions to combat the growing threat of multidrug-resistant infections in healthcare environments.

### 4.1 Limitation

This study has certain limitations, including the relatively small sample size of 32 strains, which may not capture the full genetic diversity of *blaTEM* across different environmental and clinical settings. The absence of functional validations, environmental sampling, potential biases in bioinformatic tools, and the inability to validate findings through experimental techniques. Moreover, the analysis did not capture all genetic variations or evolutionary factors influencing BlaTEM gene diversity across broader populations.

## CRediT authorship contribution statement

**Jackson Henry Katonge:** Conceptualization, Data curation, Formal analysis, Investigation, Methodology, Supervision, Validation, Visualization, Writing – original draft, Writing – review & editing. **Zainabu Khamis Ally:** Data curation, Investigation, Methodology, Writing – original draft.

## Funding

No fund was received for this study.

## Declaration of competing interest

The authors declare that have no known competing financial interests or personal relationships that could have appeared to influence the work reported in this paper.

## Data Availability

Data will be made available on request.
